# Supplementary figures and images for: Adolescent suicide attempts in Brazil and impact of COVID-19 pandemic: A temporal analysis
Source: PLOS Glob Public Health. 2026 Feb 4;6(2):e0005478. doi: 10.1371/journal.pgph.0005478 (PMC12871967; doi:10.1371/journal.pgph.0005478)

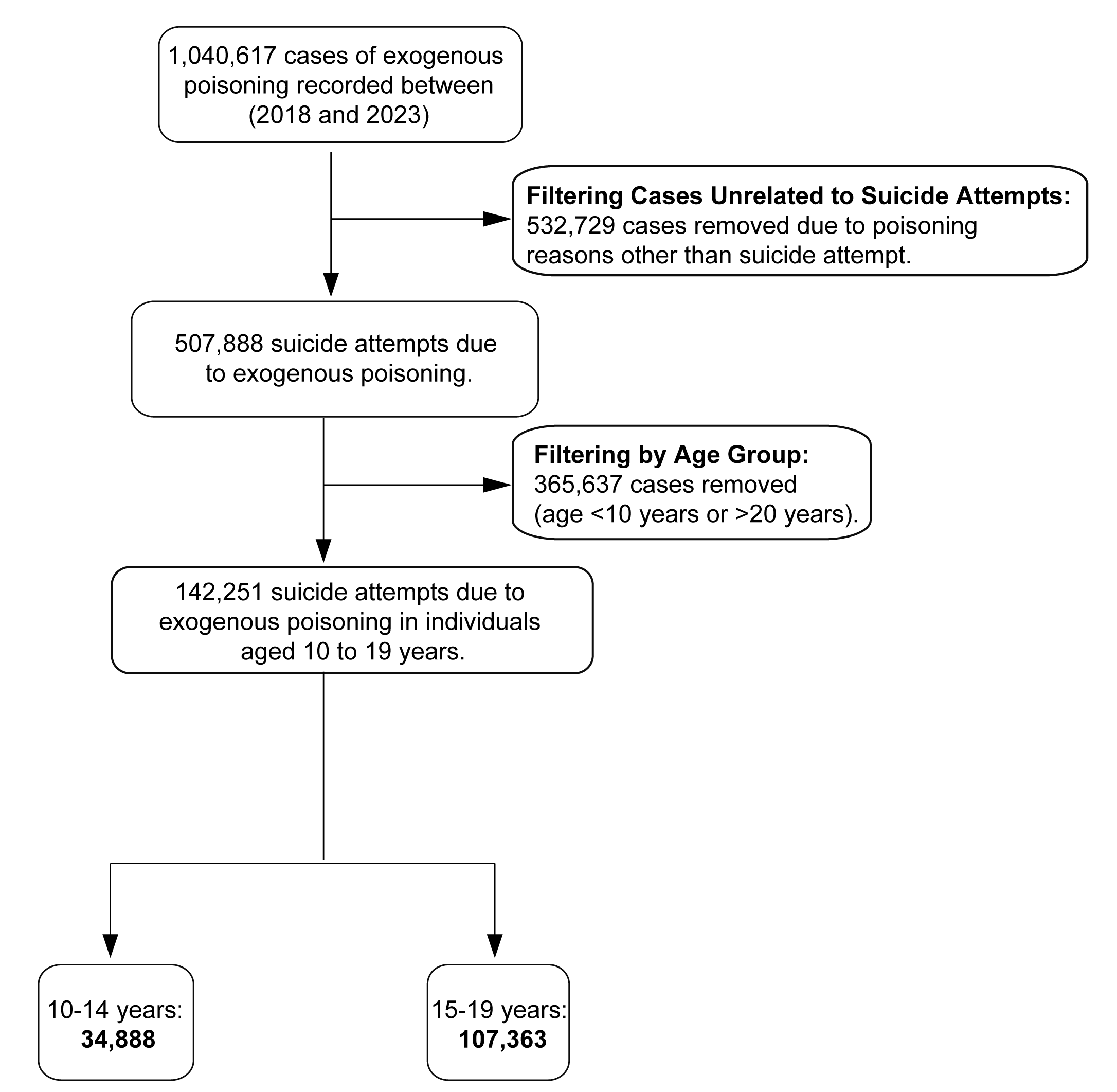

Supplement: S1 Fig — (TIF) [file pgph.0005478.s001.tif]

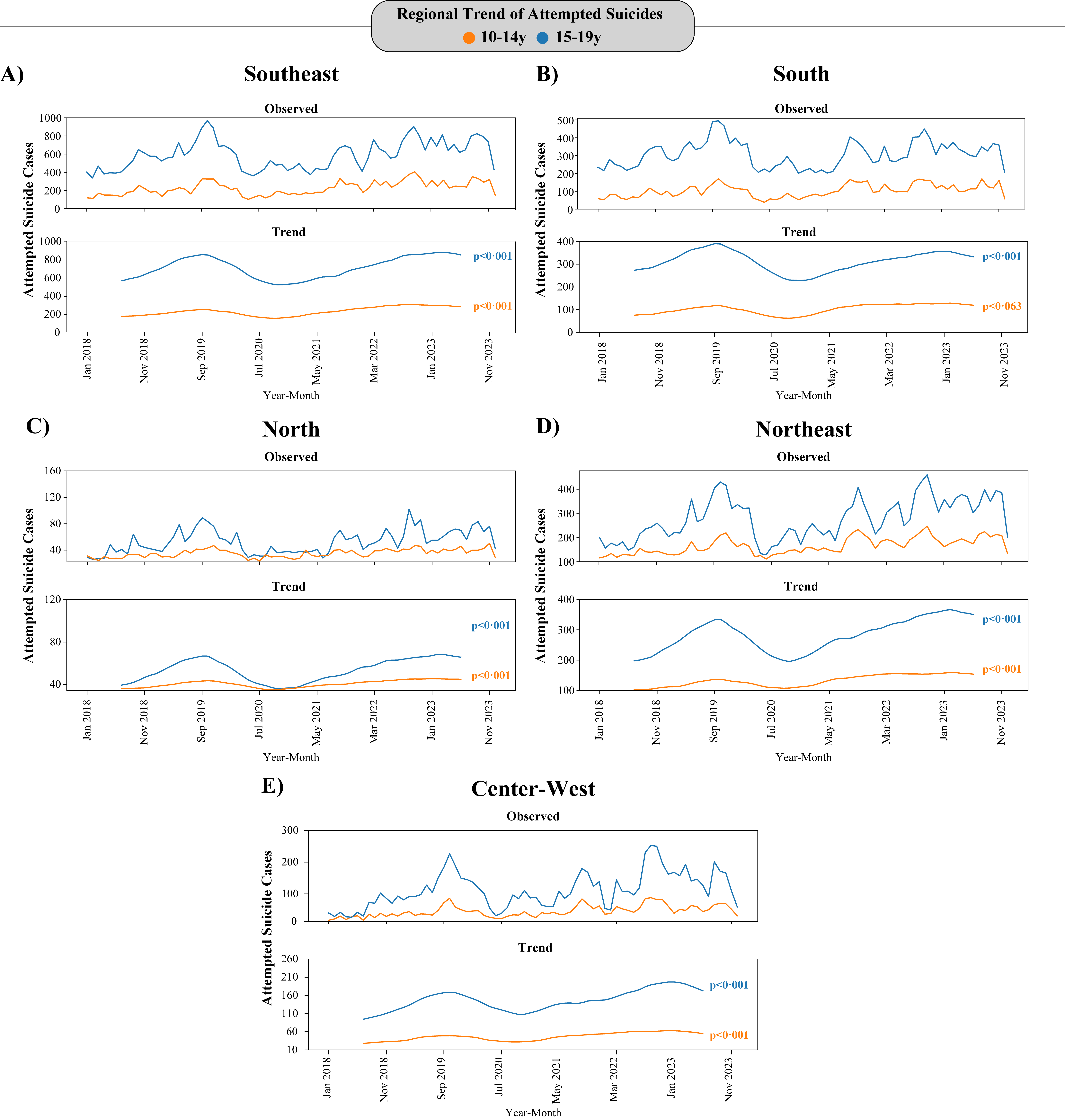

Supplement: S2 Fig — (TIF) [file pgph.0005478.s002.tif]
