# Supplementary material for: Adolescent suicide attempts in Brazil and impact of COVID-19 pandemic: A temporal analysis
Source: PLOS Glob Public Health. 2026 Feb 4;6(2):e0005478. doi: 10.1371/journal.pgph.0005478 (PMC12871967; doi:10.1371/journal.pgph.0005478)
Supplement: S1 Table — (DOCX) [file pgph.0005478.s003.docx]

**S1 Table: National** **Impact of the COVID-19 Pandemic on Suicide Attempt Notifications Among Adolescents**

| **Year** | **10-14 Age Group** | | | | **15-19 Age Group** | | | |  |
| --- | --- | --- | --- | --- | --- | --- | --- | --- | --- |
|  | **Observed Data** | **Counterfactual Values** | **Total Difference** | **Relative Difference** | **Observed Data** | **Counterfactual Values** | **Total Difference** | **Relative Difference (%)** |  |
|  |  |  |  |  |  |  |  |  |  |
| 2020 | 2738 | 4557·99[2970-6637·35] | -1819·99[-232--3899·35] | -66·47[-8·47--142·42] | 9776 | 15274·12 [10757·33-21000·89] | -5498·12 [-981·33--11224·89] | -56·24 [-10·04--114·82] |  |
| 2021 | 5997 | 5456·69[3174·77-8764·65] | 540·31[2822·23--2767·65] | 9·01[47·06--46·15] | 16261 | 18624·25 [11707·31-28259·57] | -2363·25 [4553·69--11998·57] | -14·53 [28--73·79] |  |
| 2022 | 7404 | 5270·86[2943·15-8762·16] | 2133·14[4460·85--1358·16] | 28·81[60·25--18·34] | 20349 | 18007·95 [10816·76-28396·16] | 2341·05 [9532·24--8047·16] | 11·5 [46·84--39·55] |  |
| 2023 | 6730 | 4786·35[2627·39-8049·78] | 1943·65[4102·61--1319·78] | 28·88[60·96--19·61] | 20008 | 16303·76 [9602·46-26076·42] | 3704·24 [10405·54--6068·42] | 18·51 [52·01--30·33] |  |

Table note: This table presents an interrupted time-series analysis of monthly suicide attempt notifications among adolescents aged 10-14 and 15-19 at national level, from 2020 to 2023. Observed data represent the actual reported cases, while counterfactual values estimate the expected number of cases in the absence of the COVID-19 pandemic. The total difference indicates the absolute reduction or increase in cases, while the relative difference (%) represents the percentage change compared to the counterfactual values. Negative values highlight reductions in reported attempts, suggesting potential disruptions in surveillance and reporting systems during the pandemic.
