# Supplementary material for: Adolescent suicide attempts in Brazil and impact of COVID-19 pandemic: A temporal analysis
Source: PLOS Glob Public Health. 2026 Feb 4;6(2):e0005478. doi: 10.1371/journal.pgph.0005478 (PMC12871967; doi:10.1371/journal.pgph.0005478)
Supplement: S2 Table — (DOCX) [file pgph.0005478.s004.docx]

**S2 Table: Regional Impact of the COVID-19 Pandemic on Suicide Attempt Notifications Among Adolescents**

| **Year** |  | **10-14 Age Group** | | | | **15-19 Age Group** | | | |  |
| --- | --- | --- | --- | --- | --- | --- | --- | --- | --- | --- |
|  | **Region** | **Observed Data** | **Counterfactual Values** | **Total Difference** | **Relative Difference (%)** | **Observed Data** | **Counterfactual Values** | **Total Difference** | **Relative Difference (%)** |  |
|  |  |  |  |  |  |  |  |  |  |  |
| 2020 | Centro-Oeste | 412 | 515·46 [435·25-595·67] | -103·46 [-23·25--183·67] | **-25·11 [-5·64 - -44·58]** | 4420 | 5504·33 [5148·95 - 5859·71] | -1084·33 [-728·95 - -1439·71] | **-24·53 [-16·49 - -32·57]** |  |
| 2021 | Centro-Oeste | 588 | 465·79 [369·53-562·05] | 122·21 [218·47-25·95] | 20·78 [37·15-4·41] | 6740 | 7327·36 [6739·6 - 7915·12] | -587·36 [0·4 - -1175·12] | -8·71 [0·01 - -17·44] |  |
| 2022 | Centro-Oeste | 729 | 454·19 [355·82-552·56] | 274·81 [373·18-176·44] | 37·7 [51·19-24·2] | 8244 | 5909·98 [5373·89 - 6446·06] | 2334·02 [2870·11 - 1797·94] | 28·31 [34·81 - 21·81] |  |
| 2023 | Centro-Oeste | 611 | 450·46 [351·52-549·4] | 160·54 [259·48-61·6] | 26·27 [42·47-10·08] | 7720 | 6785·81 [6137·89 - 7433·73] | 934·19 [1582·11 - 286·27] | 12·1 [20·49 - 3·71] |  |
| 2020 | Nordeste | 775 | 1101·13 [939·58-1262·68] | -326·13 [-164·58--487·68] | **-42·08 [-21·24- -62·93]** | 7478 | 12292·09 [11549·1 - 13035·08] | -4814·09 [-4071·1 - -5557·08] | **-64·38 [-54·44 - -74·31]** |  |
| 2021 | Nordeste | 1197 | 961·43 [772·28-1150·59] | 235·57 [424·72-46·41] | 19·68 [35·48-3·88] | 13072 | 15309·98 [14149·25 - 16470·72] | -2237·98 [-1077·25 - -3398·72] | -17·12 [-8·24 - -26] |  |
| 2022 | Nordeste | 1394 | 930·99 [740·19-1121·79] | 463·01 [653·81-272·21] | 33·21 [46·9-19·53] | 15692 | 12871·27 [11791·35 - 13951·2] | 2820·73 [3900·65 - 1740·8] | 17·98 [24·86 - 11·09] |  |
| 2023 | Nordeste | 1305 | 921·79 [730·75-1112·83] | 383·21 [574·25-192·17] | 29·36 [44-14·73] | 15804 | 14230·58 [12991·75 - 15469·41] | 1573·42 [2812·25 - 334·59] | 9·96 [17·79 - 2·12] |  |
| 2020 | Norte | 125 | 219·99 [174·79-265·19] | -94·99 [-49·79--140·19] | **-75·99 [-39·83- -112·15]** | 1456 | 2583·27 [2389·01 - 2777·53] | -1127·27 [-933·01 - -1321·53] | **-77·42 [-64·08 - -90·76]** |  |
| 2021 | Norte | 173 | 190·72 [140·77-240·67] | -17·72 [32·23--67·67] | -10·24 [18·63 - -39·12] | 2244 | 2859·79 [2612·36 - 3107·21] | -615·79 [-368·36 - -863·21] | -27·44 [-16·42 - -38·47] |  |
| 2022 | Norte | 240 | 181·83 [131·64-232·03] | 58·17 [108·36-7·97] | 24·24 [45·15-3·32] | 2992 | 2654·86 [2401·98 - 2907·73] | 337·14 [590·02 - 84·27] | 11·27 [19·72 - 2·82] |  |
| 2023 | Norte | 245 | 178·64 [128·46-228·82] | 66·36 [116·54-16·18] | 27·09 [47·57-6·6] | 2968 | 2654·2 [2393·03 - 2915·37] | 313·8 [574·97 - 52·63] | 10·57 [19·37 - 1·77] |  |
| 2020 | Sudeste | 1981 | 2829·56 [2556·64-3102·48] | -848·56 [-575·64--1121·48] | **-42·83 [-29·06- -56·61]** | 20837 | 27078·97 [25597·06 - 28560·87] | -6241·97 [-4760·06 - -7723·87] | **-29·96 [-22·84 - -37·07]** |  |
| 2021 | Sudeste | 2626 | 2578·71 [2236·34-2921·08] | 47·29 [389·66--295·08] | 1·8 [14·84--11·24] | 29288 | 36395·16 [33989·27 - 38801·05] | -7107·16 [-4701·27 - -9513·05] | **-24·27 [-16·05 - -32·48]** |  |
| 2022 | Sudeste | 3479 | 2497·18 [2141·07-2853·29] | 981·82 [1337·93-625·71] | 28·22 [38·46-17·99] | 38188 | 30662·77 [28483·53 - 32842·01] | 7525·23 [9704·47 - 5345·99] | 19·71 [25·41 - 14] |  |
| 2023 | Sudeste | 3130 | 2463·31 [2102·65-2823·97] | 666·69 [1027·35-306·03] | 21·3 [32·82-9·78] | 38164 | 35123·95 [32543·18 - 37704·72] | 3040·05 [5620·82 - 459·28] | 7·97 [14·73 - 1·2] |  |
| 2020 | Sul | 949 | 1436·78 [1275·36-1598·21] | -487·78 [-326·36--649·21] | **-51·4 [-34·39- -68·41]** | 10008 | 13976·36 [13290·16 - 14662·56] | -3968·36 [-3282·16 - -4654·56] | **-39·65 [-32·8 - -46·51]** |  |
| 2021 | Sul | 1413 | 1304·17 [1110·23-1498·11] | 108·83 [302·77--85·11] | 7·7 [21·43- -6·02] | 13700 | 17846·84 [16807·19 - 18886·48] | -4146·84 [-3107·19 - -5186·48] | **-30·27 [-22·68 - -37·86]** |  |
| 2022 | Sul | 1562 | 1265·31 [1066·73-1463·89] | 296·69 [495·27-98·11] | 18·99 [31·71-6·28] | 16280 | 15588·8 [14622·81 - 16554·79] | 691·2 [1657·19 - -274·79] | 4·25 [10·18 - -1·69] |  |
| 2023 | Sul | 1439 | 1251·06 [1051·15-1450·98] | 187·94 [387·85--11·98] | 13·06 [26·95- -0·83] | 15376 | 17190·24 [16097·56 - 18282·92] | -1814·24 [-721·56 - -2906·92] | -11·8 [-4·69 - -18·91] |  |

Table note: This table presents an interrupted time-series analysis of monthly suicide attempt notifications among adolescents aged 10-14 and 15-19 across different regions from 2020 to 2023. Observed data represent the actual reported cases, while counterfactual values estimate the expected number of cases in the absence of the COVID-19 pandemic. The total difference indicates the absolute reduction or increase in cases, while the relative difference (%) represents the percentage change compared to the counterfactual values. Negative values highlight reductions in reported attempts, suggesting potential disruptions in surveillance and reporting systems during the pandemic
