# Supplementary material for: Adolescent suicide attempts in Brazil and impact of COVID-19 pandemic: A temporal analysis
Source: PLOS Glob Public Health. 2026 Feb 4;6(2):e0005478. doi: 10.1371/journal.pgph.0005478 (PMC12871967; doi:10.1371/journal.pgph.0005478)
Supplement: Attachment — Submitted filename: response PGH_suicidal_V2.docx [file pgph.0005478.s006.docx]

**JOURNAL REQUIREMENTS:**

**1. Please send a completed 'Competing Interests' statement, including any COIs declared by your co-authors. If you have no competing interests to declare, please state "The authors have declared that no competing interests exist". Otherwise please declare all competing interests beginning with the statement "I have read the journal's policy and the authors of this manuscript have the following competing interests:"**

***Reply: The authors have declared that no competing interests exist. This statement has been included in the revised manuscript and confirmed in the submission form.***

**2. In the online submission form, you indicated that The data that support the findings of this study will be available upon reasonable request to the corresponding author of the study.**

**All PLOS journals now require all data underlying the findings described in their manuscript to be freely available to other researchers, either 
1. In a public repository, 
2. Within the manuscript itself, or 
3. Uploaded as supplementary information.**

***Reply:* *All data underlying the findings are derived from the publicly available SINAN database (Brazilian Information System for Notifiable Diseases), accessible at:***
[***https://datasus.saude.gov.br/informacoes-de-saude-tabnet/***](https://datasus.saude.gov.br/informacoes-de-saude-tabnet/)

***The data availability statement has been revised in the manuscript to clarify that the dataset is fully open-access, anonymized by the Ministry of Health, and compliant with PLOS’s open data policy.***

**This policy applies to all data except where public deposition would breach compliance with the protocol approved by your research ethics board. If your data cannot be made publicly available for ethical or legal reasons (e.g., public availability would compromise patient privacy), please explain your reasons by return email and your exemption request will be escalated to the editor for approval. Your exemption request will be handled independently and will not hold up the peer review process, but will need to be resolved should your manuscript be accepted for publication. One of the Editorial team will then be in touch if there are any issues.**

**3. Please provide separate figure files in .tif or .eps format.**

**For more information about figure files please see our guidelines: 
https://journals.plos.org/globalpublichealth/s/figures** 
[**https://journals.plos.org/globalpublichealth/s/figures#loc-file-requirements**](https://journals.plos.org/globalpublichealth/s/figures#loc-file-requirements)

***Reply: All figures have been exported and uploaded as separate .tif files with appropriate resolution and format according to PLOS requirements. These files are included in this resubmission.***

**4. Some material included in your submission may be copyrighted. According to PLOS’s copyright policy, authors who use figures or other material (e.g., graphics, clipart, maps) from another author or copyright holder must demonstrate or obtain permission to publish this material under the Creative Commons Attribution 4.0 International (CC BY 4.0) License used by PLOS journals. Please closely review the details of PLOS’s copyright requirements here: PLOS Licenses and Copyright. If you need to request permissions from a copyright holder, you may use PLOS's Copyright Content Permission form.**

**Please respond directly to this email or email the journal office and provide any known details concerning your material's license terms and permissions required for reuse, even if you have not yet obtained copyright permissions or are unsure of your material's copyright compatibility.**

**Potential Copyright Issues:
Figure 1: please (a) provide a direct link to the base layer of the map (i.e., the country or region border shape) and ensure this is also included in the figure legend; and (b) provide a link to the terms of use / license information for the base layer image or shapefile. We cannot publish proprietary or copyrighted maps (e.g. Google Maps, Mapquest) and the terms of use for your map base layer must be compatible with our CC-BY 4.0 license.**

**Note: if you created the map in a software program like R or ArcGIS, please locate and indicate the source of the basemap shapefile onto which data has been plotted.**

**If your map was obtained from a copyrighted source please amend the figure so that the base map used is from an openly available source. Alternatively, please provide explicit written permission from the copyright holder granting you the right to publish the material under our CC-BY 4.0 license.**

**Please note that the following CC BY licenses are compatible with PLOS license: CC BY 4.0, CC BY 2.0 and CC BY 3.0, meanwhile such licenses as CC BY-ND 3.0 and others are not compatible due to additional restrictions.**

**If you are unsure whether you can use a map or not, please do reach out and we will be able to help you. The following websites are good examples of where you can source open access or public domain maps: 
* U.S. Geological Survey (USGS) - All maps are in the public domain. (http://www.usgs.gov) 
* PlaniGlobe - All maps are published under a Creative Commons license so please cite “PlaniGlobe, http://www.planiglobe.com, CC BY 2.0” in the image credit after the caption. (http://www.planiglobe.com/?lang=enl) 
* Natural Earth - All maps are public domain. (**[**http://www.naturalearthdata.com/about/terms-of-use/**](http://www.naturalearthdata.com/about/terms-of-use/)**)**

***Reply: Figure 1 was generated using R software and publicly available Natural Earth shapefiles (public domain).***

***Source:*** [***https://www.naturalearthdata.com/about/terms-of-use/***](https://www.naturalearthdata.com/about/terms-of-use/)

***This information has been added to the figure legend in the revised manuscript. No copyrighted or proprietary material (e.g., Google Maps) has been used.***

**5. If the reviewer comments include a recommendation to cite specific previously published works, please review and evaluate these publications to determine whether they are relevant and should be cited. There is no requirement to cite these works unless the editor has indicated otherwise. 
*Reply:* *All suggested references were reviewed. Relevant citations have been incorporated into the Discussion section of the revised manuscript where appropriate.***

**6. Please review your reference list to ensure that it is complete and correct. If you have cited papers that have been retracted, please include the rationale for doing so in the manuscript text, or remove these references and replace them with relevant current references. Any changes to the reference list should be mentioned in the rebuttal letter that accompanies your revised manuscript. If you need to cite a retracted article, indicate the article’s retracted status in the References list and also include a citation and full reference for the retraction notice.**

***Reply: The reference list was rechecked for accuracy and completeness. All corrections and additions are reflected in the updated manuscript submitted with this version.***

**REVIEWER 1:**

**Reviewer #1: The Topic of the article is interesting and the results are fascinating. Authors are requested to revise as per instructions**

***Reply:* *We sincerely thank the reviewer for their thoughtful comments and valuable suggestions, which have significantly improved the clarity and depth of our manuscript. We have carefully revised the paper according to each point raised. All modifications are highlighted in track changes in the revised version.***

***Below, we provide a point-by-point response.*

1. Abstract: Define exogenous poisoning**

***Reply:* *Thank you for this comment. We added a short definition in the Abstract (lines 40-42).***

**2. Key words: Use MESH Words up to 10**

***Reply:* *We thank the reviewer for the comment. Keywords were updated to include ten MeSH terms.***

**3. Add a study settings section to methods: with focus on existing national policy, child rights, relevant health programmes, ongoing conflicts, socio political issues**

***Reply* *We thank the reviewer for the suggestion. An explanation was added, expanding the description of Brazil’s Unified Health System (SUS), child protection laws, and the Psychosocial Care Network (RAPS).***

***We also included information on the Centro de Atenção Psicossocial Infantil (CAPS I), which serves children and adolescents with severe or persistent psychological distress (including substance use–related cases), and is recommended for municipalities with populations above 70,000 inhabitants. These centers play a key role in early identification and longitudinal mental health care for youth (lines 163-177).***

**4. Tables have to be structured as per SAMPL guidelines**

***Reply:* *Thank you for the observation. Tables were reformatted following SAMPL standards.***

**5. all the recommendations in this paper are very general they should be rewritten specifically for the study area and in line with the existing health system and education department**

***Reply:* *We thank the reviewer for this valuable suggestion. The Discussion section was revised to include Brazil-specific recommendations aligned with the national health and education systems. We emphasized the integration of suicide prevention strategies into primary care and school-based mental health programs, as well as the need for intersectoral collaboration and a coordinated national pathway for adolescent mental health care. These additions make the recommendations context-specific while maintaining language suitable for an international readership.***

***(Lines 433-443)***

**6. Authors should discuss with local experts and understand how gender in the study area contributes to more suicide attempts- is it child marriage/teen pregnancy? sex abuse or violence or any other factor? Please elaborate.**

***Reply:* *We appreciate this point. However, we also clarified that the SINAN dataset does not provide variables on sexual or domestic violence, which represents a limitation of this study and highlights the need for future gender-specific analyses. (lines 414- 418)***

**7. Authors should describe why covid 19 resulted in drop in suicide - is there corporal punishment in schools? domestic violence dropped? The authors should describe this in the discussion.**

***Reply:* *We thank the reviewer for this insightful comment. We expanded the Discussion to include a more detailed explanation of possible mechanisms underlying the temporary reduction in suicide attempt notifications during the COVID-19 pandemic (lines 374–381). In Brazil, corporal punishment is prohibited in schools, but the school environment can still represent a source of psychological stress for adolescents due to bullying, social exclusion, and peer conflicts. During the lockdown period, reduced in-person school interactions may have temporarily decreased exposure to such stressors, while simultaneously limiting access to supportive services and formal notification pathways. We also clarified that the observed reduction most likely reflects underreporting and disruptions in healthcare and educational systems rather than a true decline in suicidal behavior.***

**8. Authors should connect how at each stage of adolescence, adolescents are prone for mental health risks and suicides and mention the web of causation specific to the study area.**

***Reply:* *We agree with the reviewer*. *A paragraph was added to acknowledge that due to the lack of data granularity, we were unable to analyze risk differences across early and late adolescence. This was included as a limitation, along with a note that increased screen time and reduced social interaction during the pandemic could have influenced adolescents’ mental health (Lines 411-418).***

**9. Methods and results should be rewritten in a simple manner keeping in mind the amateur readers.**

***Reply:* *We appreciate the reviewer’s suggestion and carefully revisited the Methods and Results sections to ensure clarity and coherence. However, we respectfully believe that maintaining a detailed level of technical description is essential for the reproducibility and transparency of epidemiological research. Simplifying these sections further could risk omitting critical methodological information necessary for replication. Therefore, while minor linguistic adjustments were made to improve readability, the original structure and analytical depth were preserved to maintain scientific rigor.***
